# Extracellular volume fraction mapping in the myocardium, part 1: evaluation of an automated method

**DOI:** 10.1186/1532-429X-14-63

**Published:** 2012-09-10

**Authors:** Peter Kellman, Joel R Wilson, Hui Xue, Martin Ugander, Andrew E Arai

**Affiliations:** 1 National Heart, Lung, and Blood Institute, National Institutes of Health, Bethesda, MD, USA; 2 Siemens Corporate Research, Princeton, NJ, USA; 3 Department of Clinical Physiology, Karolinska Institute and Karolinska University Hospital, Stockholm, Sweden

**Keywords:** Extracellular, Diffuse fibrosis, Edema, Late enhancement, Motion correction, Co-registration

## Abstract

**Background:**

Disturbances in the myocardial extracellular volume fraction (ECV), such as diffuse or focal myocardial fibrosis or edema, are hallmarks of heart disease. Diffuse ECV changes are difficult to assess or quantify with cardiovascular magnetic resonance (CMR) using conventional late gadolinium enhancement (LGE), or pre- or post-contrast T1-mapping alone. ECV measurement circumvents factors that confound T1-weighted images or T1-maps, and has been shown to correlate well with diffuse myocardial fibrosis. The goal of this study was to develop and evaluate an automated method for producing a pixel-wise map of ECV that would be adequately robust for clinical work flow.

**Methods:**

ECV maps were automatically generated from T1-maps acquired pre- and post-contrast calibrated by blood hematocrit. The algorithm incorporates correction of respiratory motion that occurs due to insufficient breath-holding and due to misregistration between breath-holds, as well as automated identification of the blood pool. Images were visually scored on a 5-point scale from non-diagnostic (1) to excellent (5).

**Results:**

The quality score of ECV maps was 4.23 ± 0.83 (m ± SD), scored for n = 600 maps from 338 patients with 83% either excellent or good. Co-registration of the pre-and post-contrast images improved the image quality for ECV maps in 81% of the cases. ECV of normal myocardium was 25.4 ± 2.5% (m ± SD) using motion correction and co-registration values and was 31.5 ± 8.7% without motion correction and co-registration.

**Conclusions:**

Fully automated motion correction and co-registration of breath-holds significantly improve the quality of ECV maps, thus making the generation of ECV-maps feasible for clinical work flow.

## Background

Cardiovascular magnetic resonance (CMR) using late gadolinium enhancement (LGE) provides excellent depiction of myocardial infarction (MI) and focal scar, and has become an accepted standard for assessing viability [1]. LGE is also useful to detect and characterize fibrosis that is “patchy” in appearance associated with non-ischemic cardiomyopathies such hypertrophic cardiomyopathy (HCM) [2]. Diffuse myocardial fibrosis is more difficult to distinguish using LGE since the myocardial signal intensity may be nearly isointense and may be globally “nulled” thus appearing to be normal tissue [3]. Quantitative measurement of the myocardial tissue longitudinal relaxation time constant (T1) following administration of an extracellular Gadolinium-based contrast agent is sensitive to increased extracellular volume associated with diffuse myocardial fibrosis but has limitations due to a variety of confounding factors [4,5] such as gadolinium clearance rate, time of measurement, injected dose, body composition, and hematocrit. These factors cause a significant variation in post-contrast T1 making it difficult to distinguish diseased and normal tissue based on absolute T1 values. Pre-contrast T1 varies with water content and may be elevated in cases of diffuse myocardial fibrosis. Pre-contrast T1 also varies significantly with field strength [6]. Direct measurement of extracellular volume (ECV) was initially developed for quantifying the myocardial extracellular fractional distribution volume [7] and has been proposed as a means for detection and quantification of diffuse myocardial fibrosis [3,8-13]. This approach is based on the change in T1 following administration of an extracellular contrast agent and circumvents the limitation of a single post-contrast T1 measurement in detecting a global change in T1. The myocardial ECV is measured as the percent of tissue comprised of extracellular space, which is a physiologically intuitive unit of measurement and is independent of field strength [6]. ECV has been shown to correlate with collagen volume fraction [9,10]. This topic is of current interest as a diagnostic tool for non-ischemic cardiomyopathies as well as for understanding aging processes [9,11-13]. The myocardial ECV may also be elevated in edema [14] and may have benefits for quantitative assessment as compared to simple T1 and T2 measurements.

Current methods of generating ECV maps require cumbersome off-line post processing [13]. The goal of this study was to develop and evaluate an automated method for producing a pixel-wise map of ECV that would be adequately robust for clinical work flow. The benefits of pixel-wise mapping over more commonly used ROI based measurements is the ability to directly visualize the spatial extent and severity of inhomogeneities in ECV in relation to anatomical boundaries. A major challenge is the correction of motion that occurs due to inconsistent breath-holding during the acquisition of images and due to patient movement between breath-holds. The proposed method uses a new method for motion correction of a series of inversion recovery images with widely varying contrasts that is used for T1-mapping [15], and incorporates a new scheme for co-registration of pre-and post-contrast T1-maps.

## Methods

### ECV measurement

The ECV in the myocardium may be estimated from the concentration of extracellular contrast agent in the myocardium relative to the blood in a dynamic steady state [7,9,13].

(1) $$ECV=\left(1-hematocrit\right)\frac{\left(\frac{1}{{T1}_{myo\;post}}-\frac{1}{{T1}_{myo\;pre}}\right)}{\left(\frac{1}{{T1}_{blood\;post}}-\frac{1}{{T1}_{blood\;pre}}\right)}$$

since the change in relaxation rate ΔR1 (where R1 = 1/T1) between pre and post contrast is directly proportional to the Gd-DTPA concentration, ΔR1 = γ [Gd-DTPA] (γ = 4.5 L mmol^-1^ sec^-1^). A dynamic steady state exists for tissues which have a contrast exchange rate with the blood which is faster than the net clearance of contrast from the blood [7]. A dynamic steady state between the plasma and interstitium may be achieved by slow intravenous infusion [3,9], or by imaging 15 min following an intravenous bolus administration [12,13] for normally perfused myocardium, although 15 min may not be adequate for recently infarcted myocardium [16]. The bolus method was used in this study since it fits well with clinical workflow and permits conventional late enhancement imaging at the desired dose. The ECV formula (Eq [1]) implies that our myocardial ECV measurements include both the intra- and extravascular space, and is related to the estimate of extracellular extravascular volume fraction (Ve) [8,12] which includes additional constant factors. The factor (1-hematocrit), which varies between individuals, represents the blood volume of distribution (blood ECV) and converts the equation from a partition coefficient calculation to a myocardial ECV.

### Image acquisition

T1-mapping is based on inverting the magnetization and acquiring images at different times during the magnetization recovery. The acquisition is ECG triggered and all images are acquired at the same cardiac phase in late diastole using a Modified Look Locker Inversion Recovery (MOLLI) approach [17]. This method has been validated [17,18]. Methods for T1-measurement such as cine inversion recovery (IR) [8,19] which acquire the measurements of magnetization at multiple cardiac phases may be used for measuring the T1 in the myocardium but are not suitable for mapping with pixel resolution. Following respiratory motion correction (described below), T1-fitting of the data at each pixel is performed by a non-linear least square fit to the exponential recovery curve abs(A – B·exp(−TI/T1*)), where TI is the measured inversion time for each acquired image, the absolute value (abs) is used since the images are magnitude detected. T1* is the effective time constant which includes the effect of the image readout [17] related to the desired T1 as T1 = T1* (B/A – 1). This “Look-Locker” correction used in MOLLI [17] was derived analytically [20] for the case of FLASH readout with continuous RF, whereas in the case of MOLLI is being applied to gated SSFP readout. This leads to bias errors in T1 [21] which are reduced by using a relatively low excitation flip angle. Imaging was performed on 1.5 T Siemens Avanto and Espree scanners (Siemens Medical Solutions, Erlangen, Germany), equipped with 45 mT/m and 200 mT/m/s, and 33 mT/m and 170 mT/m/s gradient systems, respectively. The study protocol typically included acquiring T1-maps for 2 slices (mid-ventricular short axis and four chamber long axis views) both pre-contrast and approx. 15–20 min following intravenous administration of 0.15 mmol/kg Gd-DTPA. Both pre- and post-contrast T1-maps were acquired with the same imaging parameters. The original published MOLLI protocol [17] acquired 11 images over 17 heartbeats. The MOLLI imaging protocol used in this study acquired data at 8 inversion times over an 11 heart beat breath-hold at end-expiration with 2 inversions. The initial protocol used acquired 3 images acquired after the first inversion, 3 heart beat pause, and 5 images acquired after the second inversion. The protocol was later modified to reduce heart rate variability by acquiring 5 images after the first inversion, followed by a 3 heartbeat pause and then acquire 3 images after the second inversion. Both protocols (3–5 and 5–3) acquired 8 images in 11 heartbeats.

Typical imaging parameters were: non-selective inversion pulse, steady state free precession single shot read out with 35° excitation flip angle, field of view 360 × 270 mm^2^, slice thickness 6 mm, minimum inversion time 110 ms, inversion time increment 80 ms, matrix 192×130, voxel size 2.1 × 1.9 × 6.0 mm^3^, TR/TE 2.4/1.0 ms, 7/8 partial Fourier plus parallel imaging factor 2 with 140 ms readout imaging duration. The maximum inversion time was approximately 5 sec at 60 bpm. The protocol has subsequently been revised for increased spatial resolution using a matrix of 256×144, TR/TE 2.7/1.1 ms (200 ms readout imaging duration), with voxel size 1.9 × 1.4 × 6.0 mm^3^ for heart rates < 90 bpm, and 192×130 for hearts above 90 bpm.

### T1-Mapping and motion correction (MOCO)

**Figure 1:**
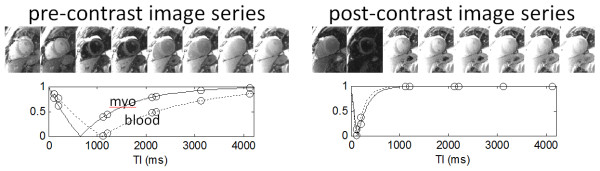
Pre- and post-contrast image series acquired using a Modified Look Locker Inversion Recovery (MOLLI) sequence used for T1-mapping.

Although patients are instructed to hold their breath (typically 11 s) during the MOLLI acquisition of images used for T1-mapping, residual respiratory motion frequently introduces artifacts due to diaphragmatic drift or difficulty with the breath-hold. If uncorrected, this motion may lead to errors in the pixel-wise estimation of T1 thereby degrading the image quality of the T1-maps. The unique challenge inherent to registration of inversion recovery images acquired at different inversion times is the large variation in image contrast (Figure 1). Furthermore, different tissue species with differing values of T1 such as blood, fat, normal myocardium, edematous myocardium, and infarcted myocardium will experience signal nulling at different inversion times. Partial volume cancellation at the boundaries between pixels of different T1s will occur when the 2 species are out-of-phase which further confounds the image registration problem. The difference in contrast and appearance between images is so large that simple pairwise image registration using either intensity or mutual information based metrics is inadequate.

In this study we used the novel motion correction algorithm described by Xue, et al. [15] based on using motion-free synthetic images presenting similar contrast to the original acquired inversion recovery images. Additionally, ECV maps were generated from motion corrected T1-maps after co-registration and blood pool measurements as described in following sections. Using the synthetic image registration approach, the difficulty of handling large variations of image contrast throughout inversion recovery is bypassed. Motion correction is done with an iterative approach based on initial T1 estimates and the generation of a series of motion free synthetic inversion recovery images which are used at each inversion time for registration with the measured MOLLI images. The initial T1-map is estimated using images acquired at the shortest and longest values of TI, which results in a crude estimate of T1 sufficient to initialize the iterative process. It was determined that although the contrast between the short and long TI images is not identical, these images were suitable for registration since they avoided the problem of tissue nulling and partial volume cancellation. From the initial estimate of T1 at each pixel, synthetic images are calculated exploiting the known exponential form of inversion recovery. Synthetic images are calculated for each of the measured images using the estimated values of T1. The synthetic images are all at a fixed respiratory position and, importantly, each synthetic image has similar contrast to the corresponding measured image, making them suitable for intensity based image registration. Due to the non-rigid nature of tissue deformation caused by respiratory motion, a non-rigid image registration algorithm based on the classical optical flow method was to estimate the motion deformation [15]. Image warping for both MOCO and co-registration utilized a 5^th^-order B-spline interpolator in order to minimize loss of spatial resolution.

Following motion correction, the T1 map is generated by a pixel-wise curve fitting using a three parameter signal model for MOLLI inversion recovery. The downhill simplex minimization algorithm was used [15]. At each pixel the sum-of-squares of the residual errors of the measured data to the exponential model fit was calculated as a goodness of fit map.

### Co-registration

**Figure 2:**
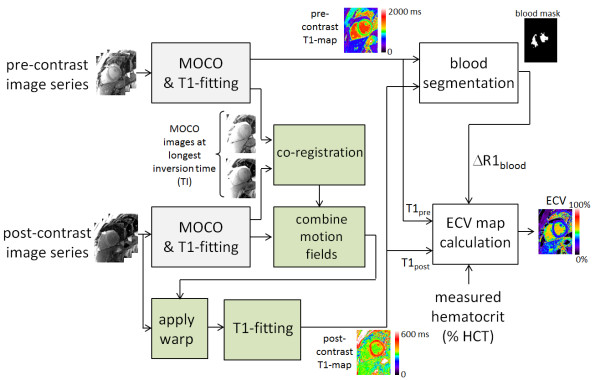
**Schematic flow chart for automatic generation of ECV maps by respiratory motion correction of pre- and post-contrast inversion recovery image series, co-registration of each series with each other, and blood pool segmentation.** Note that color scales are different for pre and post contrast examples.

Pre- and post-contrast image series are acquired in separate breath-holds which are typically 15–30 minutes apart. Even small differences in respiratory position or changes in patient position due to movement will cause significant misregistration of the images. Co-registration of the pre-and post-contrast images is performed (Figure 2) using a non-rigid image registration applied to the longest inversion time image of the already motion corrected images. In this way, the magnetization of the long inversion time images is almost completely recovered and the image contrast between pre- and post-contrast series will be very similar thereby facilitating intensity based image registration methods. Co-registration is achieved by applying the estimated motion deformation field to the post-contrast series. Rather than simply applying the deformation (warping) to the T1-map or to the already motion corrected image series, a composite deformation using the original respiratory motion fields of the post-contrast series combined with the deformation from the co-registration step is applied a single time to avoid an unnecessary loss of spatial resolution. The final T1-map for the post-contrast series is calculated by pixel-wise fitting.

The complete processing workflow was fully automated. Once the user specifies the study, the software retrieves all images for appropriate paired series of pre and post contrast MOLLI acquisitions at the same slice location, performs all processing, and produces T1-maps and ECV maps in dicom format which may be pushed to the PACS. The processing time in the current implementation is approximately 45 sec per ECV series including pre- and post-contrast T1-mapping with MOCO and co-registration, and blood pool segmentation.

### Blood pool segmentation

**Figure 3:**
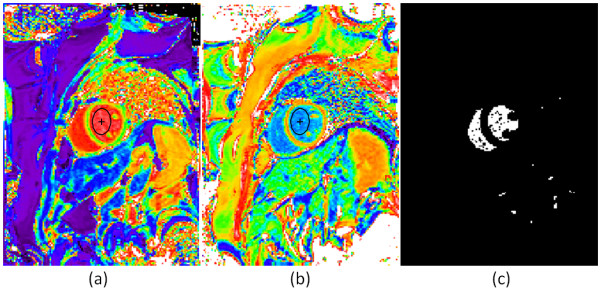
**Example T1-maps acquired pre- (left) and post- (center) contrast and automatic segmentation mask (right) used to estimate T1-values of blood for ECV calculation.** Manually drawn blood pool ROIs were used for comparison with automatic segmentation estimates.

Calculation of the ECV (Eq [1]) requires knowledge of the change in blood relaxation rate (ΔR1_blood_). An automatic method determining a blood region was developed based on the pre-contrast T1-map. The pre-contrast T1 of blood is in the range 1300–1800 ms which is greater than the T1 of most tissues. A binary mask was created from the pre-contrast T1 map using a threshold of 1250 ms. Isolated pixels which might be attributed to noise were removed, and the resultant mask was “eroded” to remove edge pixels that might correspond to blood-tissue boundaries with intermediate values due to partial volume effects (Figure 3). The values for pre- and post-contrast blood T1 were calculated as the median of all values of the T1-map identified by the mask. The post-contrast T1-map was, as a result of the co-registration, located in the same position as the pre-contrast T1-map. The change in blood relaxation rate was subsequently calculated as ΔR1_blood_ = 1/T1_post_ – 1/T1_pre_. Automatic calculation of blood T1’s were compared with values from manually drawn ROIs in N = 50 subjects.

### Scoring

**Figure 4:**
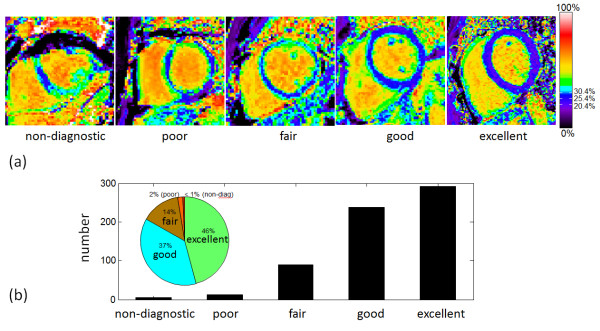
**Quality of ECV maps was scored on a scale of 1–5 for N = 600 maps across 338 subjects.** ECV map images are provided as examples for each quality category.

ECV maps were scored for overall quality based on a 5 point scale. N = 600 maps corresponding to 338 patients imaged consecutively were scored. The rating was based on image artifacts, delineation of myocardial blood pool borders, and homogeneity of myocardial signal. A score of 5 (excellent) was assigned when the myocardial borders were crisp and distinct from the blood pool with less than or equal to 1 pixel of partial volume border and no noticeable geometric distortion. The myocardial signal had to be homogeneous (uniform ECV) in areas assessed to be normal tissue (i.e., no focal scar on late enhancement). A score of 4 (good) was assigned when one of the above criteria was absent, i.e., there were 1–2 pixels of representative myocardial ECV with slightly increased border between blood and myocardium, or geometric distortion were present but not impacting the ECV values, or there were small regions (10%) of inhomogeneity in the myocardium. A score of 3 (fair) meant that that 2 criteria under the excellent category were absent, i.e., only a thin stripe of myocardium was present from which to sample the representative ECV, or geometric distortion was present, or multiple regions had inhomogeneity deemed to be artifactual but affected less than 50% of the myocardium. A score of 2 (poor) meant that inhomogeneities or geometric distortion affected greater than 50% of the myocardium making it difficult to distinguish regions of normal myocardium from noise or artifacts, but that ECV sampling was still possible in isolated areas. A score on 1 (non-diagnostic) meant that ECV could not be measured reliably anywhere in the myocardium. Examples of ECV maps in each scoring categories are shown in Figure 4(a). It is important to note that in for certain pathologies, the partial volume at the blood myocardium border may be mistaken for pathology or vice-versa, as emphasized in the Discussion. For the purpose of the scoring the quality of maps, the readout involved a judgment based on comparison of all the maps including with and without motion correction and/or co-registration, as well as LGE images and patient history.

Performance of the motion correction and co-registration of T1-maps were scored separately in addition to the overall ECV map quality. These sub-scores were assessed as follows. Pre-contrast T1-maps were compared before and after MOCO, and were assessed as to whether the MOCO T1-map was better, the same, or worse than the T1-map without MOCO. Post-contrast T1-maps with and without MOCO were similarly scored. The basis of assessment was the improvement of myocardial borders and reduction of artifacts. Performance of the co-registration of pre- and post-contrast data was similarly scored according to better, same, or worse by comparing the ECV maps but with and without the step of co-registration.

### Human subject protocol

ECV imaging was performed between Sept 2009 and May 2011, 952 patients referred for CMR assessment of known or suspected heart disease prospectively underwent ECV imaging. Hematocrit was measured from a venous blood sample drawn just prior to the CMR study. This study was approved by the local Institutional Review Boards of the National Heart, Lung, and Blood Institute and Suburban Hospital, and all subjects gave written informed consent to participate. A total of 1680 ECV maps were generated by automated off-line processing as described above (728 studies imaged matching short and long axis views both pre- and post-contrast, and the 224 remaining studies only acquired a single slice). Scoring of ECV map quality was performed on 600 maps (338 subjects) acquired consecutively, and statistics for automatic blood measurements were made based on the complete set. Additionally, cine MRI of cardiac function and phase sensitive inversion recovery (PSIR) late Gadolinium enhancement imaging [22] was performed on all subjects.

### ECV measurement

For the purpose of establishing the value of ECV in “normal” myocardium, subjects were categorized as “normal” if they were without known significant systemic illness, demonstrated normal biventricular systolic function, chamber size, and wall thickness, absent significant valvular dysfunction, were assessed clinically as LGE negative and did not have apparent edema as evidenced by elevated pre-contrast T1 [23]. Subjects with diabetes or hypertension were excluded. In order to establish ECV values for normal myocardium, ROIs for normal subjects were drawn in a mid-wall region of the myocardium to avoid contamination from partial volume with the blood pool. Values were measured on ECV maps with and without motion correction/co-registration.

### Statistics

Data are presented as mean and standard deviation. Differences between groups were tested by the paired or unpaired *t*-test as appropriate. Differences in variance were tested by the F test. Statistical significance was defined as p < 0.05.

## Results

### Blood measurements

The measured hematocrit was 41.2 ± 4.8% (N = 952 subjects) and ranged from a minimum of 18% to a maximum of 65%. A comparison was performed between the estimates of blood pool T1 made by the automatic blood pool segmentation and manually drawn ROIs (Figure 3) for N = 50 subjects. The correlation between T1-estimates using manual vs automatic blood pool segmentation was r = 0.95 (P < 0.001) for pre-contrast and r = 0.99 (P < 0.001) for post-contrast acquisitions. The pre-contrast T1 was 1473.2 ± 61.8 ms and 1478.1 ± 64.7 ms (p = 0.07), using automatic and manual measurement respectively. The post-contrast T1 was 321.9 ± 43.5 ms and 320.9 ± 42.6 ms (p = 0.10), using automatic and manual measurement respectively.

### Quality scores

**Figure 5:**
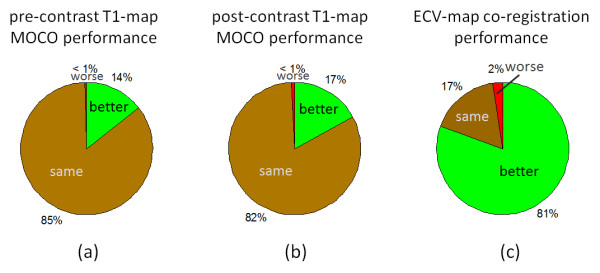
Performance of motion correction of T1-maps and co-registration for ECV-maps was scored as to whether or not there was visually detectable improvement or degradation.

**Figure 6:**
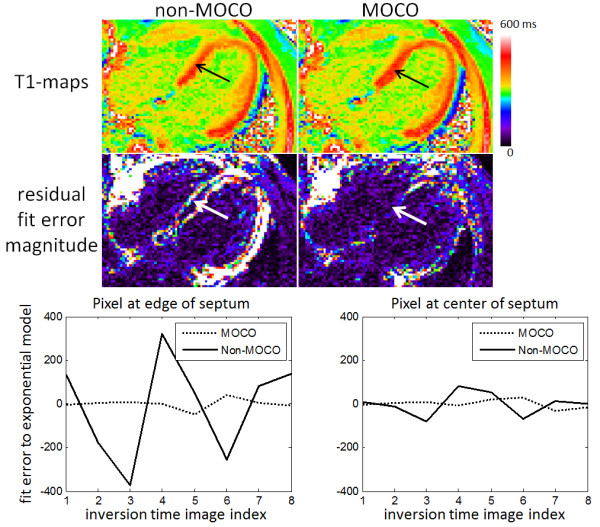
**Example case of post-contrast T1-maps acquired with significant respiratory motion which is significantly improved by MOCO, particularly in the septal region (arrows).** Residual errors from exponential model fit (arbitrary signal intensity units) are plotted versus inversion time for a pixel at the edge of the septum (left) and a pixel at the center of the septum (right) before (non-MOCO) and after MOCO.

**Figure 7:**
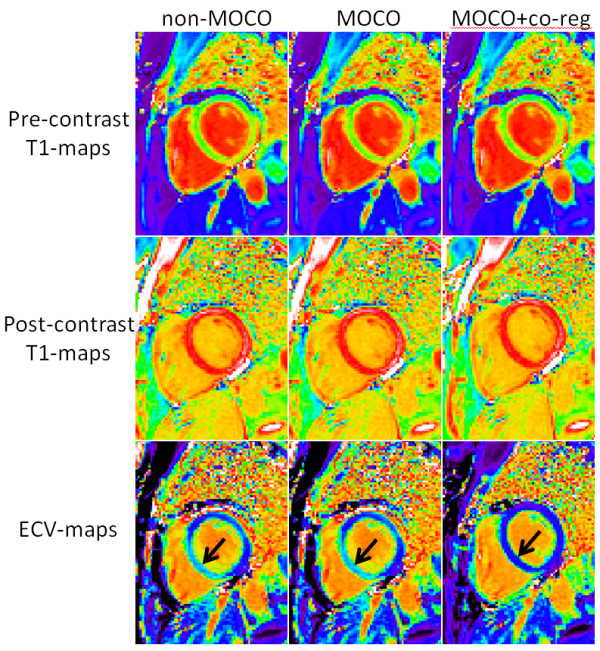
**Example case for which co-registration of data between pre- and post-contrast T1-maps acquisitions was critical.** In these examples, MOCO co-registration of the respective maps markedly improved the ECV map, eliminating an artifactually high ECV in the inferior septum (arrows) in the non-co-registered maps. MOCO did not improve the quality of individual (pre- and post-contrast) T1-maps for this example, however MOCO + co-registration was significantly improved.

The quality of ECV maps was 4.23 ± 0.83 based on N = 600 maps corresponding to 338 unique, consecutive subjects. This corresponded to 271 excellent, 225 good, 87 fair, 12 poor, and 5 non-diagnostic (Figure 4(b)). T1 maps with motion correction were assessed to be better (14%), the same (85%), and worse (<1%) for pre-contrast image series as compared to without correction, and similarly were better (17%), the same (82%), and worse (<1%) for post-contrast (Figure 5). A small number of instances arose in which the MOCO resulted in significant geometric distortion leading to mis-registration which worsened the overall score. An example of a case where MOCO improved the T1-map is shown in Figure 6 comparing the non-corrected (left) and corrected (right) map, and corresponding maps of T1-fitting error. The T1-fitting error is plotted versus the inversion time for a representative edge pixel and a midmural pixel to further illustrate the improvement after motion correction. Co-registration of the pre-and post-contrast images improved the ECV maps in 81% of the cases (example shown in Figure 7), were the same in 17%, and were worse in 2% of the cases.

### ECV measurements

**Figure 8:**
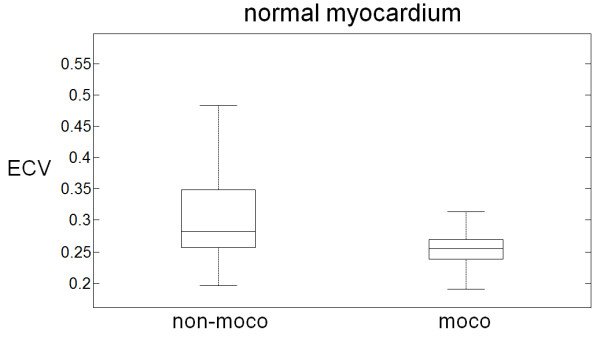
**Measured ECV values (n = 62 subjects) for normal myocardium measured in the mid-wall of the myocardium with and without motion correction and co-registration showing that motion correction significantly reduces variability (p < 0.001).** Box and whisker plots show median, 25 and 75 percentiles, and range.

Patient characteristics and risk factors for the 62 subjects used to define the range of normal values are listed in Table 1. ECV of normal myocardium was 25.4 ± 2.5% using motion correction and co-registration measured in the mid-wall for 62 subjects which were LGE negative and appeared clinically normal. Without motion correction or co-registration the apparent ECV values were 30.8 ± 7.8% (P < 0.001 compared to motion correction and co-registered). The ECV measurements without motion correction and co-registration had a greater variation due to contamination with blood or other tissue (7.8% vs 2.5%, P < 0.001, Figure 8). The ECV of normal myocardium with motion correction had a normal range (mean ± 2SD) which was 20.4-30.4%.

**Table 1:** Patient characteristics and risk factors for the 62 subjects used to define normal values

**Age**	**43.6 ± 17.4**
Gender	30 male, 32 female
Family History of CAD	10
Smoking	1 current / 13 ex-smokers
High Cholesterol	14
Hypertension	0
Diabetes	0
BMI (kg/m^2^)	26.5 ± 4.6
Creatinine (mg/dL)	0.88 ± 0.19
LVEF (%)	61.7 ± 5.9
LVEDV (mL)	143.9 ± 32.9
LVEDV index (mL/m^2^)	76.8 ± 12.2
LV mass (g)	87.4 ± 26.2
LV mass index (g/m^2^)	46 .2 ± 9.5
Wall Thickness (mm)	7.5 ± 1.8

*BMI* = Body Mass Index, *LV* = left ventricular, *EF* = ejection fraction, *EDV* = end-diastolic volume. Wall thickness was measured in the basal anteroseptum.

## Discussion

### Benefits of pixel-wise ECV mapping

ECV mapping appears promising to complement LGE imaging in cases of more homogenously diffuse myocardial disease states which affect the myocardial extracellular space. The ability to display ECV maps in quantitative units that may be interpreted on an absolute scale offers the potential for simplified detection and measurement of the extent of abnormalities affecting the myocardial ECV. There is a continuous spectrum of spatial heterogeneity between diffuse and focal fibrosis. The use of pixel-wise mapping offers a new tool to better assess the heterogeneity of the myocardial tissue and to measure ECV of the diseased regions.

### Limitations

The ability to distinguish partial volume border pixels and correctly determine whether pixels are contaminated by partial volume effects, or are true pathophysiology such as subendocardial or subepicardial fibrosis, is a residual and important issue. This issue is not only important in terms of the visual readout, but may introduce biases into quantitative measurements which become particularly significant in the context of more subtle diseases. One approach to reducing the bias in measurements is to restrict the measurement to the mid-wall region, although one must exercise caution for subjects with thin walled myocardium or patients with thin rims of subendocardial fibrosis.

The proposed approach to generating ECV maps resulted in excellent to good image quality maps in 83% of the cases. A number of factors contributed to cases of poor quality images. In some cases patient movement resulted in the slices being substantially different in appearance despite the fact that the same slice position was prescribed. Co-registration is by design only capable of correcting for in-plane motion and cannot compensate for through plane motion, although it may appear to partially compensate. In other cases, it is clear that the pre- and post-contrast images are at substantially different cardiac phases due to significant change in heart rate, despite adjusting the trigger window. Both of these factors might be improved at the time of acquisition by more careful inspection but this would complicate the workflow.

The interpretation probably can be improved by examining the pre-and post-contrast T1-maps for consistent appearance of position and cardiac phase as a quality control step. Furthermore, it is expected that the use of ECV maps will be in conjunction with pre-contrast T1-maps and conventional late enhancement images, and not read independently. As such, the ability to assess and quantify diffuse fibrosis in ECV images should provide increased diagnostic confidence since LGE image quality is generally excellent and will not share the same artifacts. Finally, the use of residual error maps (Figure 7) offers a potential as another quality metric to increase the confidence for a given region. In other words, if the error map indicates that the motion correction is high quality (low error), then the ECV values are more accurate.

Partial volume effects affect the measurement of ECV. Partial volume effects are a result of limits in in-plane spatial resolution as well as slice thickness and are observed at tissue borders such as for pixels between blood and myocardial tissue. Partial volume effects also affect the measurement of fine structures such as MI contributing to a larger variability. These same factors are also a limitation in LGE. These effects are less of a factor for measurements of globally diffuse disease.

The issue of achieving a “dynamic steady state” using a bolus [12,13] rather than slow infusion [3,9] has been studied by several researchers, however, the accuracy of this assumption and the corresponding impact on quantitative ECV measurements is unknown. In particular, it has been noted that steady state may take longer to achieve in acute myocardial infarction [16], therefore further work is required to investigate this limitation.

Despite these issues, ECV maps may be readily incorporated into the clinical workflow and may provide diagnostic information where other methods are limited. It is not intended to replace existing methods such as LGE which are excellent at depicting focal lesions, but rather to be used in concert with other techniques. When ECV is abnormally elevated, it may not be clear whether this is due to fibrosis or edema which may be either diffuse or focal. In such instances, pre-contrast T1 or T2 maps, in addition to patient history and contextual imaging clues like signs of heart failure, may help to differentiate these mechanisms.

The presence of intra-myocardial fat may affect the estimates for ECV and has not been studied. In cases of fat with low fat fraction, voxels with partial volume of fat and myocardial tissue may lead to errors in T1-mapping which assume a mono-exponential model for single T1-species. Furthermore, fat is not in fast chemical exchange with the Gadolinium contrast agent and will not experience the same T1 shortening. Multi-echo fat water separated inversion recovery may be used for late enhancement [24,25] but is challenging to incorporate into a T1-mapping sequences. Fat water separated imaging may also be used in the combined assessment to identify the presence of intra-myocardial fat.

In this study, the generation of ECV maps was performed off-line and retrospectively. In order to wholly automate the generation of ECV images on the scanner at the time of imaging, hematocrit would either have to be entered manually or automatically queried from a clinical database. These options are potentials for improvement which remain to be explored in order to make the generation of ECV images wholly automatic on the scanner as a part of the clinical work flow. While it is possible to compute a partition coefficient map without using the value of hematocrit [26], the variability in hematocrit (18.3% to 65% in this series) makes the partition coefficient less useful for measuring subtle changes in ECV expected for diffuse fibrosis.

The method for automatic segmentation of the blood pool was effective for mid-ventricular short axis or long axis views acquired in this study. For volumetric coverage which would include apical slices with a reduced size of the region of blood, it might be necessary to adapt this method to use blood values acquired at other slices with the proviso that they must be acquired in close time proximity such that the blood T1 remains the same.

### T1-mapping accuracy and precision

The accuracy and precision of T1-mapping using the MOLLI method is affected by a number of factors including the protocol, the sequence design, patient specific factors related to heart rate and blood flow, and system adjustments such as center frequency and shim. The MOLLI method for T1-mapping is widely use and a number of adaptions to the protocol have been introduced to shorten the acquisition time. Further optimization the sequence and of pre- and post-contrast T1-mapping protocols based on the MOLLI strategy is the subject of ongoing research.

A shortened acquisition acquiring 7 images in 9 heartbeats (known as ShMOLLI) [27] may be used to further reduce the breath-hold duration and achieve a heart rate independent T1 measurement by discarding samples for tissue with longer T1. The 11 heart beat protocol used in this study achieves a reliable T1 measurement by using a longer recovery between inversions without eliminating data. At longer T1’s the 9 heart beat ShMOLLI protocol uses only 5 images for T1-fitting, whereas the proposed motion corrected 11 heart beat protocol uses all 8 acquired images. The increased number of images reduces the fitting error.

In terms of the sequence design, we have recently determined that imperfect inversion efficiency leads to an underestimation of T1, representing the largest source of systematic error. This error may be greatly reduced by means of an improved inversion pulse design as well as by applying an appropriate correction. These corrections were not available at the time of this study. As a result, the normal values for T1 are underestimated by as much as 5-10%. However the systematic bias in T1-measurement results in only a small overestimation of ECV, estimated on the order of 1% or less in ECV units. This does not alter the main conclusions of this present study on the utility of ECV maps.

## Conclusions

While LGE is an excellent method for detection of focal scarring as encountered in myocardial infarction and numerous other cardiomyopathies, ECV mapping appears promising to augment LGE imaging in cases of more homogenously diffuse disease. The fully automated implementation lends itself to incorporation of ECV mapping directly into a clinical workflow. Motion correction and co-registration of breath-holds have been demonstrated to significantly improve the image quality of ECV maps.

## Abbreviations

CMR, Cardiovascular Magnetic Resonance; ECV, Extracellular volume fraction; LGE, Late gadolinium enhancement; MOCO, Motion correction; MOLLI, Modified Look-Locker inversion recovery; TI, Inversion time; ROI, Region-of-interest; PSIR, Phase sensitive inversion recovery.

## Competing interests

Dr. Arai is a principal investigator on a US government Cooperative Research And Development Agreement (CRADA) with Siemens Medical Solutions (HL-CR-05-004).

## Authors’ contributions

PK conceived of the study, developed algorithms and software, performed processing and analysis, and drafted the manuscript. JRW performed analysis of image quality. XH developed motion correction software. MU and AEA participated in design of the study. All authors participated in revising the manuscript and read and approved the final manuscript.
